# Supplementary material for: GD2 and its biosynthetic enzyme GD3 synthase promote tumorigenesis in prostate cancer by regulating cancer stem cell behavior
Source: Sci Rep. 2024 Jun 12;14:13523. doi: 10.1038/s41598-024-60052-3 (PMC11169677; doi:10.1038/s41598-024-60052-3)
Supplement: Supplementary file 1 — Supplementary Information 1. [file 41598_2024_60052_MOESM1_ESM.docx]

GD2 and its biosynthetic enzyme GD3 synthase promote tumorigenesis in prostate cancer by regulating cancer stem cell behavior

Aaqib M. Bhat^1,2.*^, Bhopal C. Mohapatra^2,8,*,#^, Haitao Luan^1^, Insha Mushtaq^1,3^, Sukanya Chakraborty^1,2^, Siddhartha Kumar^1^, Wangbin Wu^1^, Samikshan Dutta^4^, Matthew D. Storck^1^, Micah Schott^4,8^, Jane L. Meza^5,8^, Subodh M. Lele^3,8^, Ming-Fong Lin^4^, Leah M. Cook^3,8^, Eva Corey^6^, Colm Morrissey^6^, Donald W. Coulter^7,9^, M. Jordan Rowley^2,8^, Amarnath Natarajan^1,8^, Kaustubh Datta^4,8^, Vimla Band^2,8,#^, Hamid Band^1,2,3,4,8,#^

^1^Eppley Institute for Research in Cancer and Allied Diseases, University of Nebraska Medical Center, Omaha, NE; ^2^Department of Genetics, Cell Biology and Anatomy, College of Medicine, University of Nebraska Medical Center, Omaha, NE; ^3^Departments of Pathology & Microbiology, College of Medicine, University of Nebraska Medical Center, Omaha, NE; ^4^Department of Biochemistry and Molecular Biology, University of Nebraska Medical Center, Omaha, NE; ^5^Department of Biostatistics, College of Public Health, University of Nebraska Medical Center, Omaha, NE; ^6^Department of Urology, University of Washington, Seattle, WA; ^7^Department of Pediatrics, University of Nebraska Medical Center, Omaha, NE; ^8^Fred & Pamela Buffett Cancer Center, University of Nebraska Medical Center, Omaha, NE.

***Co-First Authors**

**Running Title**: GD2 in prostate cancer tumorigenesis

**Keywords**: GD2, GD3 Synthase, prostate cancer, castration-resistant prostate cancer, metastasis, cancer stem cells

**Financial support:** This research was funded by Pilot grants from the Fred & Pamela Buffett Cancer Center (HB & VB); Department of Defense grants W81XWH-17-1-0616 and W81XWH-20-1-0058 to HB and W81XWH-20-1-0546 to VB; the NIH grants R21CA241055 and R03CA253193 to VB; the NIH Pathway to Independence Award R00 GM1287671 and the NIH MIRA award R35 GM147467 (to M.J.R.); the Raphael Bonita Memorial Fund; and support to UNMC core facilities from the NCI Cancer Center Support Grant (P30CA036727) awarded to Fred & Pamela Buffett Cancer Center and from the Nebraska Research Initiative. AMB, SC and IM received the University of Nebraska Medical Center Graduate Student Fellowships.

**^#^Corresponding authors:** Hamid Band, MD, PhD, Eppley Institute for Research in Cancer and Allied Disease, 986805 Nebraska Medical Center, Omaha, NE 68198-6805, USA; Email: [hband@unmc.edu](mailto:hband@unmc.edu); Phone: 402-559-8572

Bhopal C. Mohapatra, PhD, Department of Genetics Cell Biology & Anatomy, University of Nebraska Medical Center, 985805 Nebraska Medical Center, Omaha, NE, 68198, USA. [bmohapat@unmc.edu](mailto:bmohapat@unmc.edu); Phone: 402-559-8542

Vimla Band, PhD, Department of Genetics Cell Biology & Anatomy, University of Nebraska Medical Center, 985805 Nebraska Medical Center, Omaha, NE, 68198, USA; Email: [vband@unmc.edu](mailto:vband@unmc.edu); Phone: 402-559-8572

**Conflict of interest disclosure statement:** Dr. H. Band and Dr. V. Band received funding from Nimbus Therapeutics for an unrelated project.

**Word count**: Abstract - 212, Manuscript - 4909 **Figures**: 06; **Table**: 01;

**Supplementary** **Figures**: 08

**
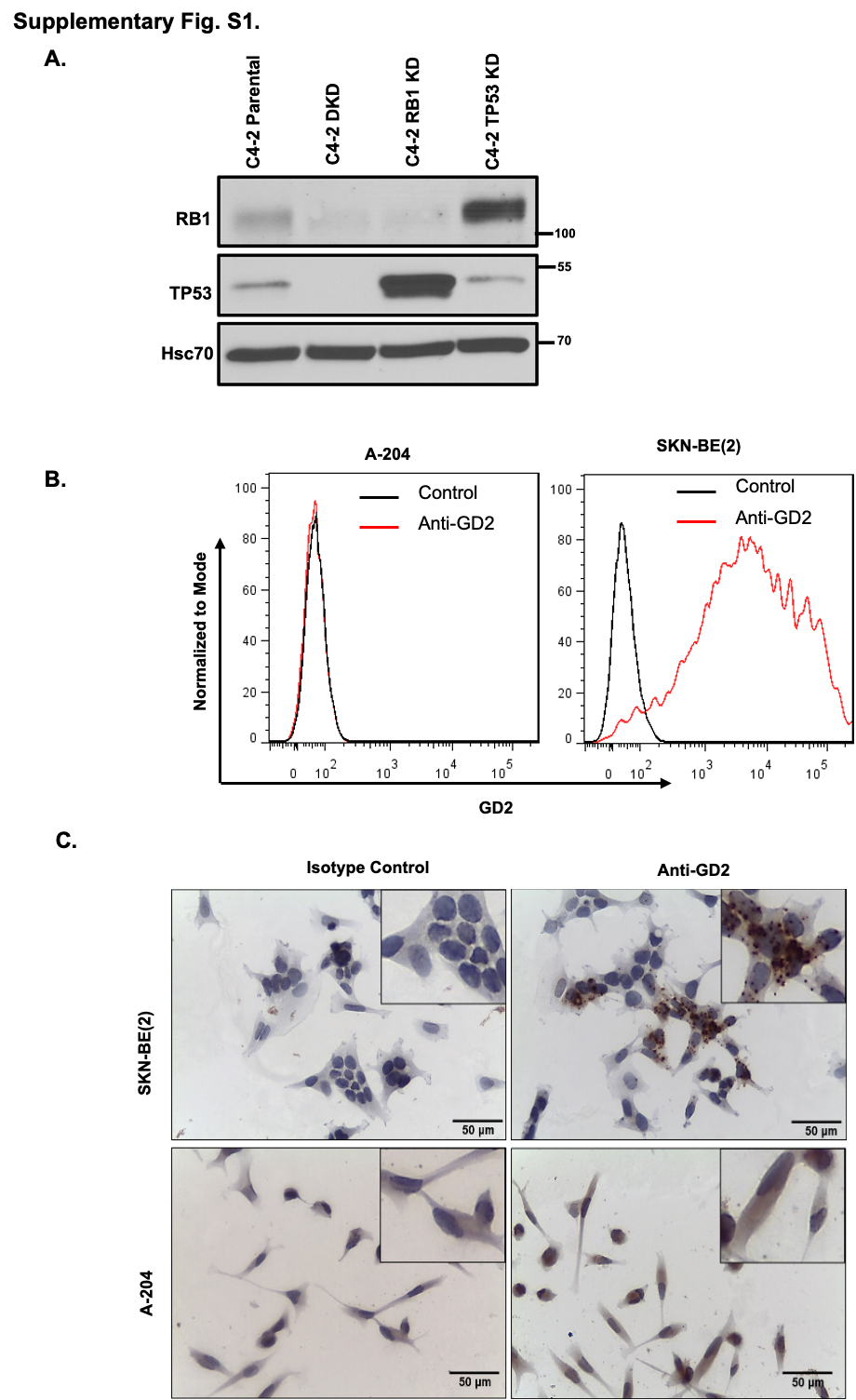
**

**Fig. S1. Confirmation of RB1, TP53 KD and RB1/TP53 double KD (DKD) in individual and DKD variants of C4-2 and validation of anti-GD2 antibody. (A)** Western blotting shows the reduction of RB1, TP53 in respective knockdown cells and both RB1 and TP53 KD in DKD samples using indicated antibodies. HSC-70 used as a loading control.  **(B)** FACS analysis of GD2^-^ A-204 Rhabdomyosarcoma and GD2^+^ SK-N-BE(2) human neuroblastoma cell lines stained with anti-GD2 vs. isotype control. **(C)** Immunohistochemistry (IHC) staining of A-204 and SK-N-BE(2) cell lines with anti-GD2 or isotype control. Scale bar, 50 µm. Insets show a higher magnification of regions within each image. GD2 antibody selectively stains the SK-NB-2 but not the A-204 cell line.

**
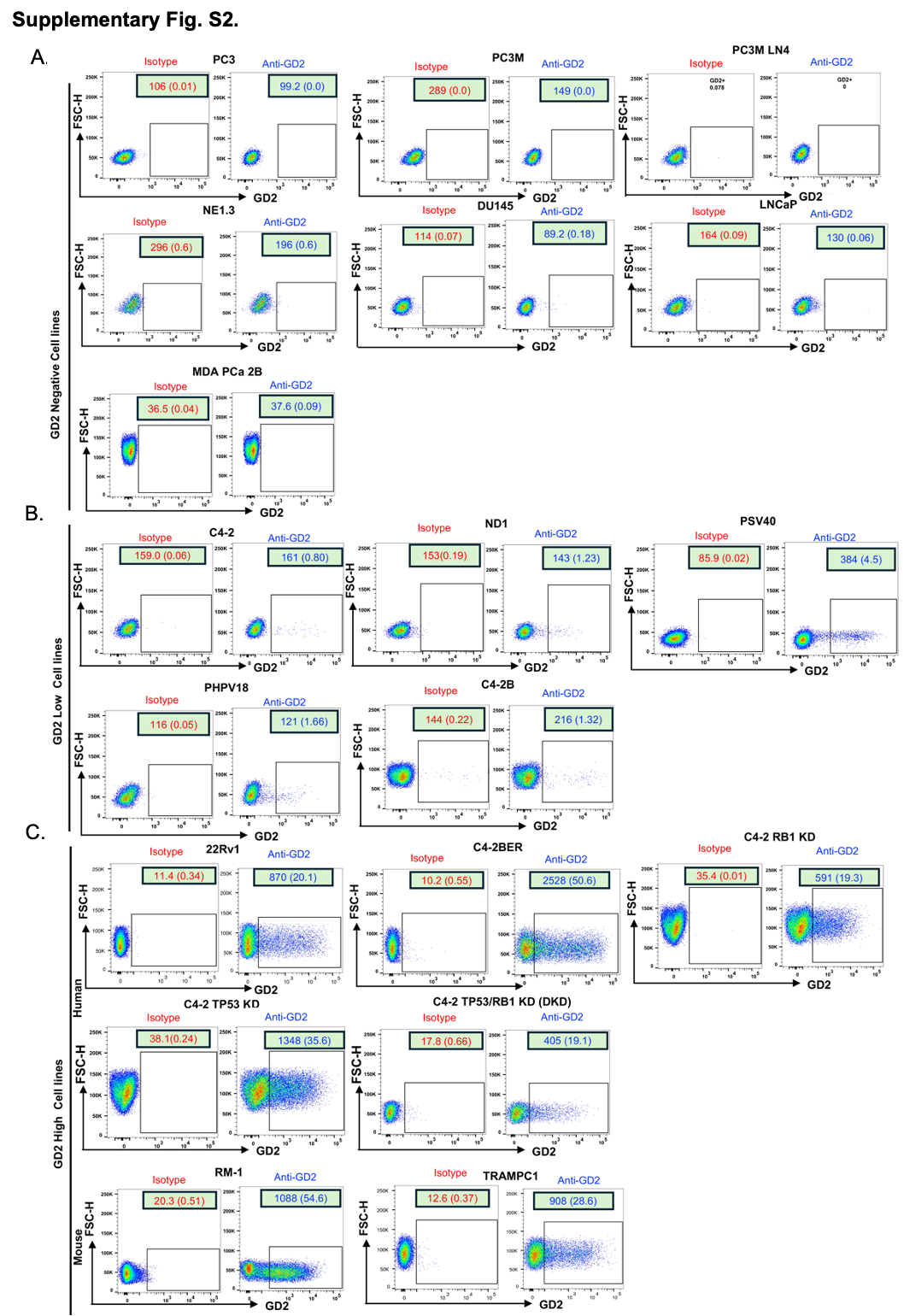
**

**Fig. S2: FACS plots of cell surface GD2 expression on human and mouse prostate cancer (PC) cell lines.** The indicated human and mouse prostate cancer cell lines were live cell stained using anti-GD2 vs. isotype control and analyzed by FACS. GD2, blue; isotype control, red. Percentage % population and MFI are indicated in FACS dot plots. (**A**) Cell lines with essentially undetectable GD2 (all human). (**B**) GD2^low^ PC cell lines (all human). (**C**) GD2^high^ human (22Rv1 and C4-2BER) and mouse PC cell lines (RM-1 and Tramp-C1) are shown.


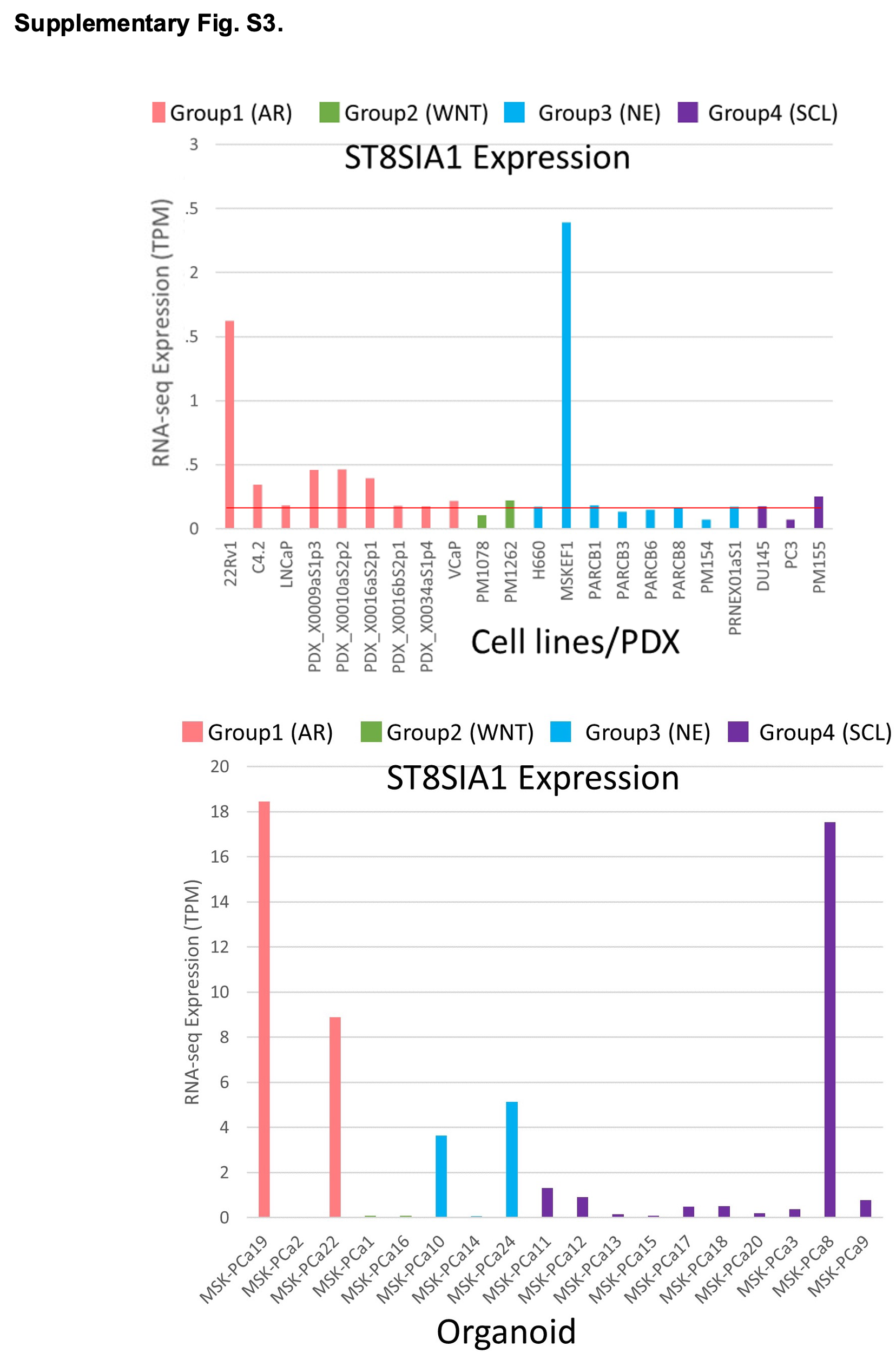


**Fig. S3: High *ST8SIA1* (GD3S) mRNA expression in a substantial proportion of CRPC patient-derived xenograft (PDX) tumors and organoid models.** Publicly available transcriptomic data published by Tang et al.[1] (GEO accession GSE199190) on CRPC PDX and cell lines (Top panel) and organoid models (Bottom panel) were queried for *ST8SIA1* mRNA expression. The CRPC groups are as per Tang et al: Group 1, Androgen Receptor dependent (CRPC-AR, peach); Group 2, Wnt dependent (CRPC-WNT, green); Group 3, neuroendocrine (CRPC-NE, blue); and Group 4, stem cell like (CRPC-SCL, purple). Gene expression is shown as normalized transcripts per million (TPM). The horizontal red line in left panel demarcates the negative/positive cutoff based on GD2^-^ LNCaP cells.

**Supplementary Fig. S4.**

**
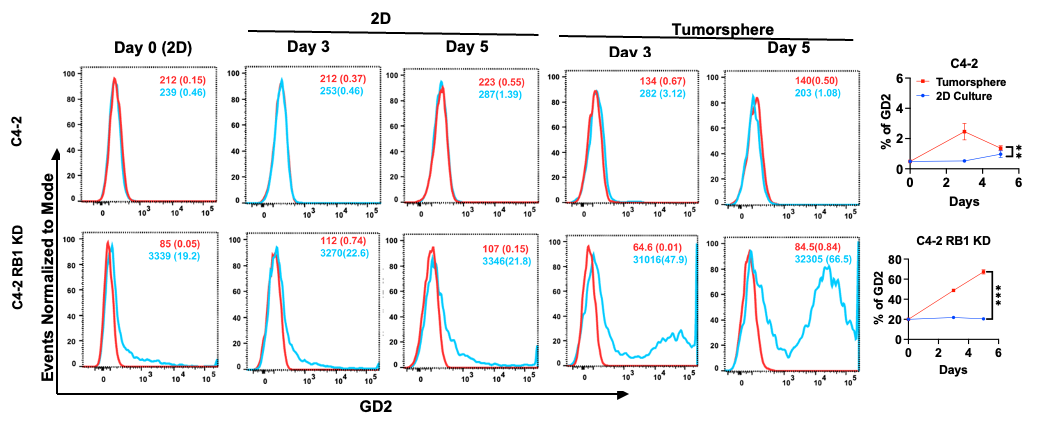
**

**Fig. S4: Enrichment of GD2^high^ cells in tumorspheres of CRPC cell lines:**

Enrichment of GD2^high^ cells in tumorspheres of CRPC cell lines. The C4-2 parental and RB1 KD cells were cultured in regular two-dimensional (2D) cultures or were seeded in tumorsphere cultures. Cells harvested at the indicated times were analyzed for cell surface GD2 expression (vs. isotype control) at the indicated times. % GD2^+^ cells and MFI are indicated. **Left**, representative FACS images; **Right**, quantitation of data. Mean ± SEM with two-way ANOVA test, **, *P* < 0.01; ***, *P* < 0.001**.**

**Supplementary Fig. S5**

**Fig. S5: Thin Layer Chromatography (TLC) immunoblotting using ganglioside extracts demonstrate presence of GD2 in parental RM-1 and 22Rv1 cells and loss in GD3S KO cells. (A)** Isolated gangliosides from indicated cell lines were resolved for TLC in silica plates. Mouse neuroblastoma cell lines, 9464D and 975A2 were used as positive and negative control for GD2 respectively. Purified GD2 obtained commercially was used as a positive standard. Represented Phosphomolybdic acid (PMA) staining of the resolved TLC plate show migration of GD2 in standard and faint comigration of GD2 in RM1, 22Rv1 and positive control 9464D cells and absence in GD3S KO and GD2 negative lanes. **(B)** TLC plate run in parallel with indicated cell lines were processed for immunoblotting with anti-GD2 antibody. GD2 immunoreactivity was observed in GD2 standard along with parental RM-1, 22Rv1 and positive control cell line 9464D.

**
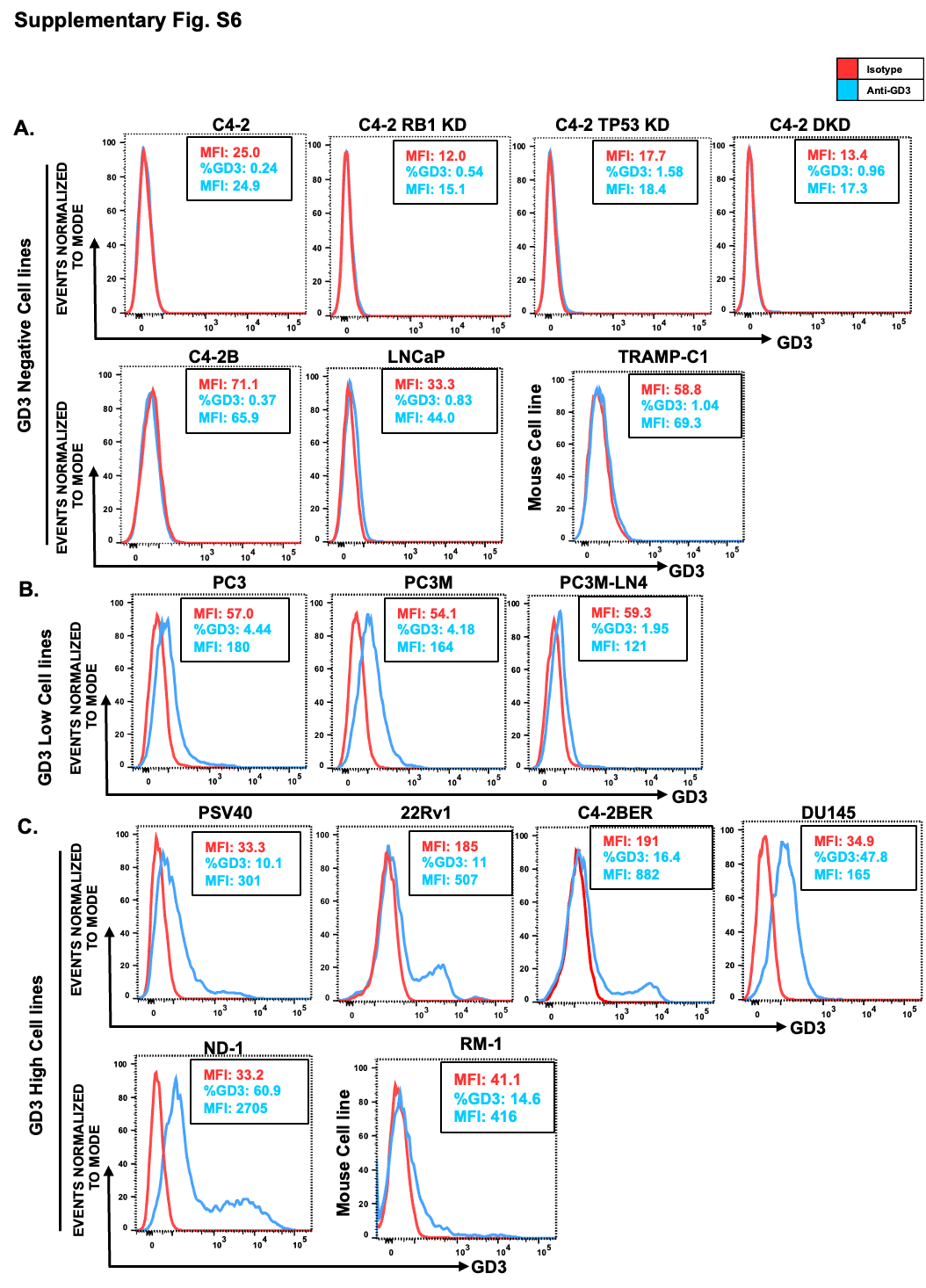
Fig. S6: FACS plots of cell surface GD3 expression on human and mouse prostate cancer (PC) cell lines.** The indicated human and mouse prostate cancer cell lines were live cell stained using anti-GD3 vs. isotype control and analyzed by FACS. GD3, blue; isotype control, red. Percentage % population and MFI are indicated in FACS dot plots. (**A**) Cell lines with essentially undetectable GD3. (**B**) GD3^low^ PC cell lines (all human). (**C**) GD3^high^ human (PSV40, 22Rv1, C4-2BER, DU145, ND-1) and mouse PC cell lines (RM-) are shown.

**
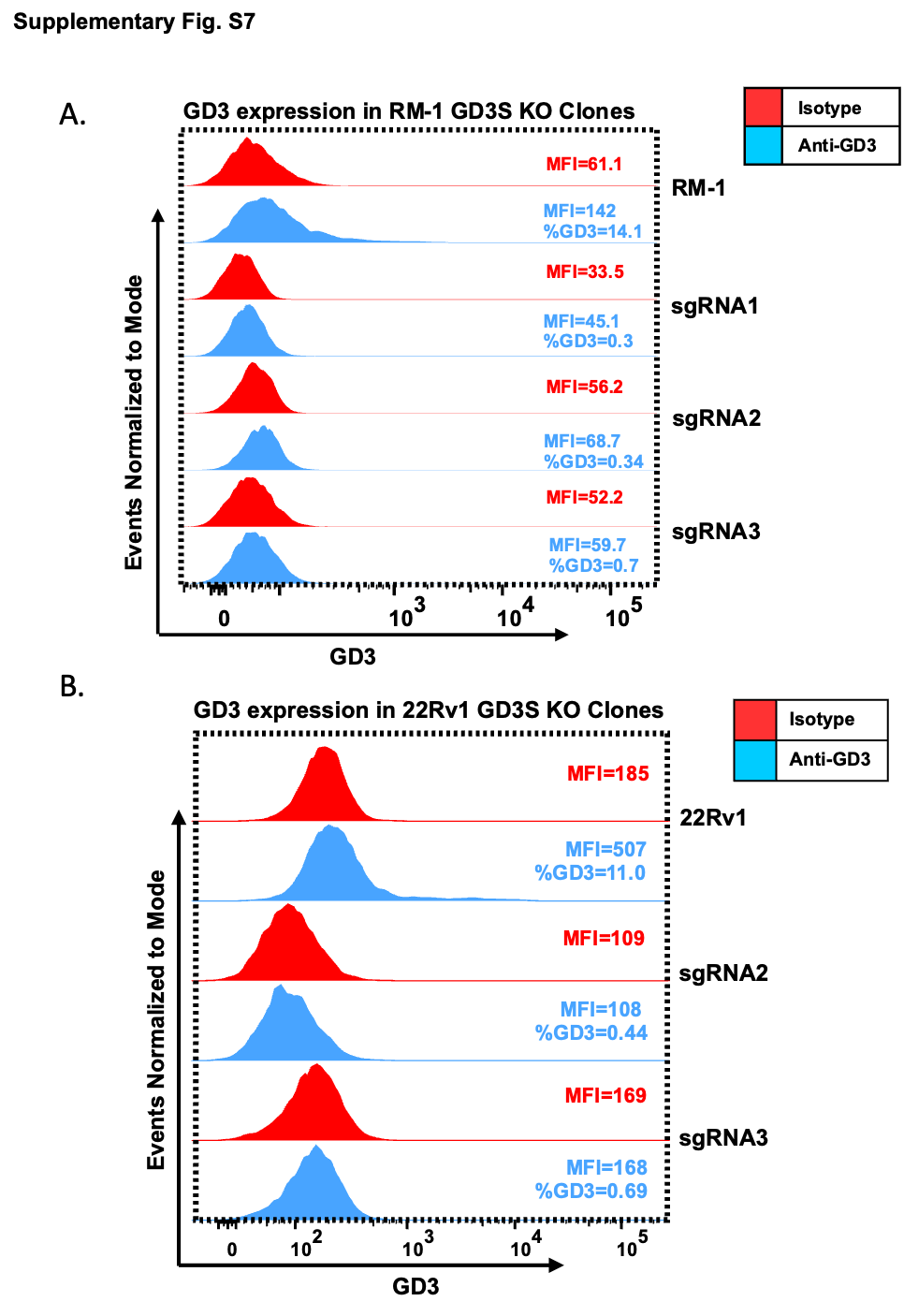
**

**Fig. S7: GD3 expression in GD3S knockout RM-1 cells.** Live cell FACS analysis of murine RM-1 and its GD3S KO variants (sgRNA1, sgRNA2 & sgRNA3) **(A)** and human 22Rv1 and its GD3S KO variants (sgRNA2 & sgRNA3) **(B)** show decreased expression of GD3 synthesis in the GD3S KO variants and is displayed as over lay FACS plots. The percentage of GD3 and MFI are indicated in FACS plots. The names of the cell lines and antibodies are mentioned on the right side of the FACS plots.

**
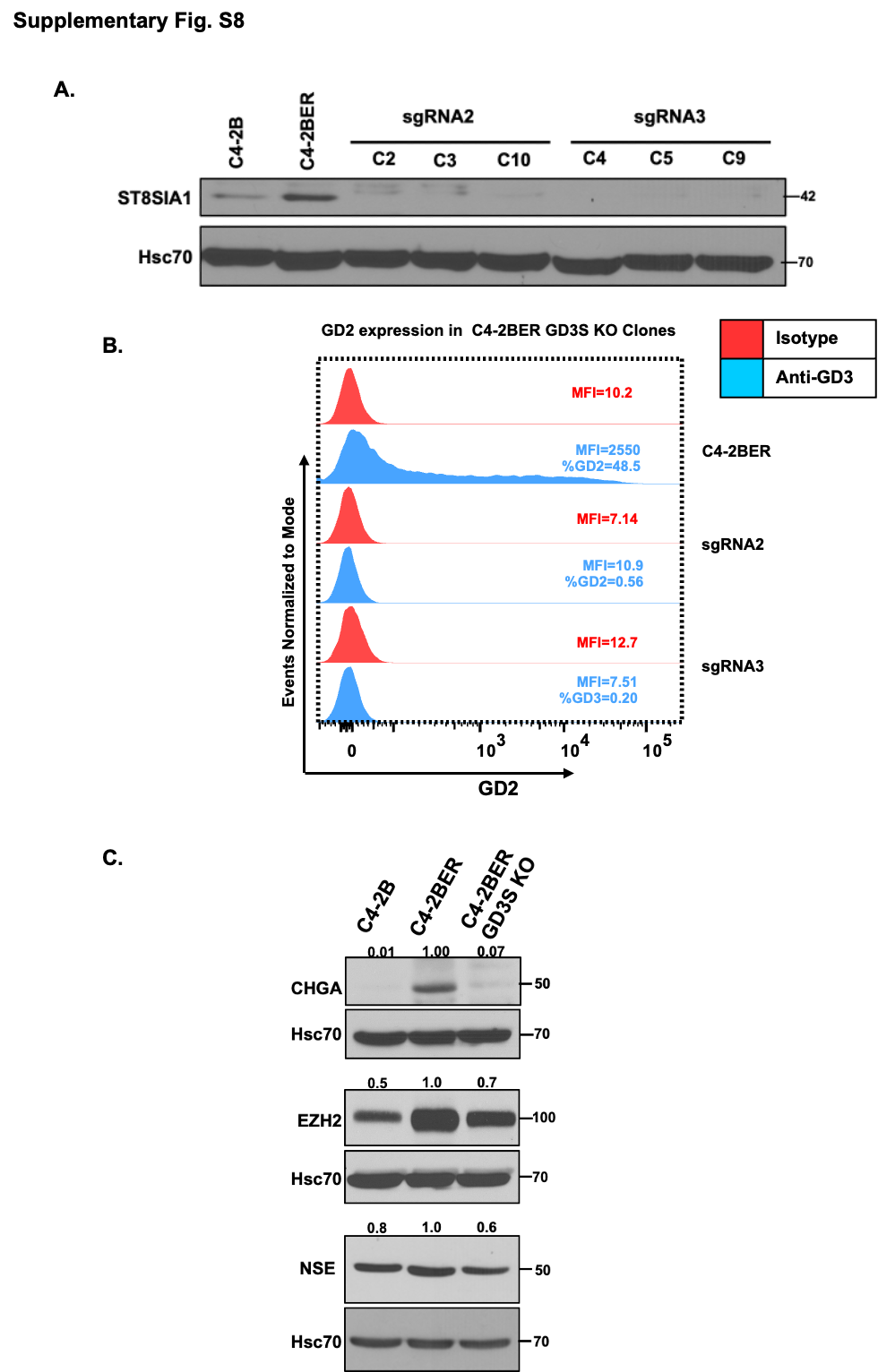
**

**Fig. S8: GD3S KO in C4-2BER cells demonstrate reduction of neuroendocrine markers:**

**(A)** Generation of GD3S knockout of RM-1 and 22Rv1 CRPC cell lines. Cells were transduced with lentiviral All-in-One CRISPR/Cas9 constructs and stable clones analyzed by Western blotting with anti-GD3S antibody; Hsc70, loading control. **(B)** Live cell FACS analysis of human C4-2BER and its GD3S KO variants (sgRNA2 & sgRNA3) show decreased expression of GD2 in the GD3S KO variants and is displayed as over lay FACS plots. The percentage of GD2 and MFI are indicated in FACS plots. The names of the cell lines and antibodies are mentioned on the right side of the FACS plots. **(C)** Representative Western blotting shows the increased neuroendocrine (NE) differentiation markers CHGA, EZH2 and NSE in enzalutamide resistant C4-2BER cells and loss of upregulated NE markers in GD3S KO variant of C4-2BER. Densitometries are indicated on the top of the bands after normalizing with HSC-70. Three independent experiments were performed.

**REFERENCES:**

1. Tang F, Xu D, Wang S, Wong CK, Martinez-Fundichely A, Lee CJ, Cohen S, Park J, Hill CE, Eng K, Bareja R, Han T, Liu EM, Palladino A, Di W, Gao D, Abida W, Beg S, Puca L, Meneses M, de Stanchina E, Berger MF, Gopalan A, Dow LE, Mosquera JM, Beltran H, Sternberg CN, Chi P, Scher HI, Sboner A, Chen Y and Khurana E (2022) Chromatin profiles classify castration-resistant prostate cancers suggesting therapeutic targets. Science 376:eabe1505. doi: 10.1126/science.abe1505
