# Supplementary material for: GD2 and its biosynthetic enzyme GD3 synthase promote tumorigenesis in prostate cancer by regulating cancer stem cell behavior
Source: Sci Rep. 2024 Jun 12;14:13523. doi: 10.1038/s41598-024-60052-3 (PMC11169677; doi:10.1038/s41598-024-60052-3)

Fig. 4 A

RM-1 GD3S KO Confirmation

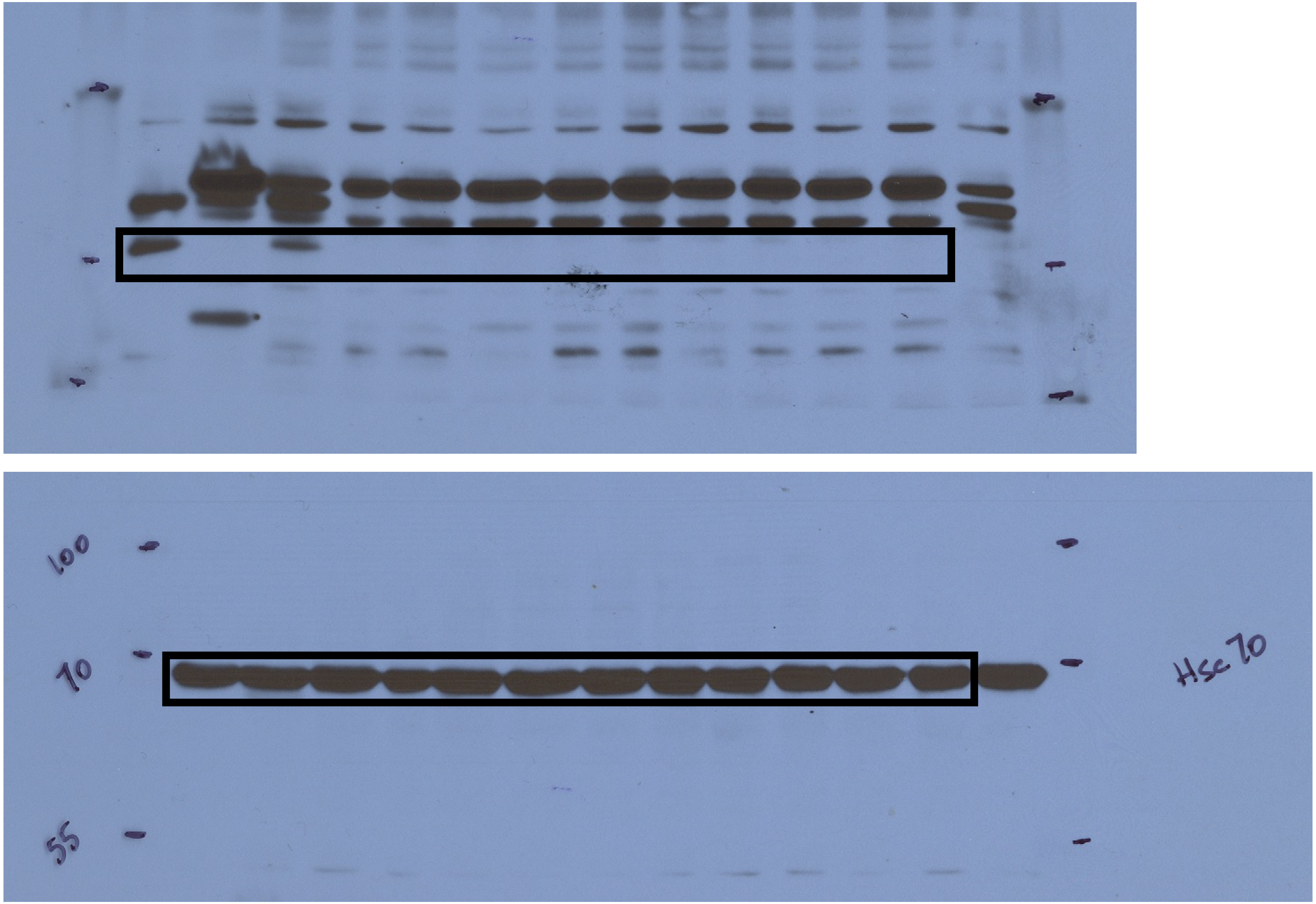

Fig. 4 B

22Rv1 GD3S KO Confirmation

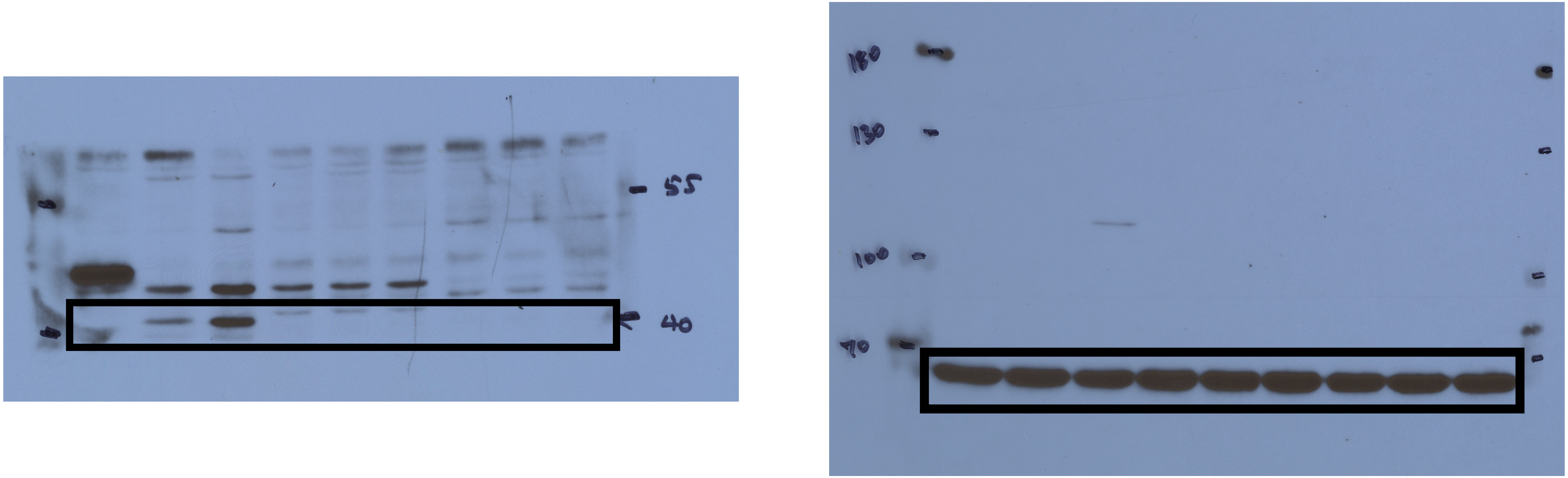

Fig. 5 E

Snail

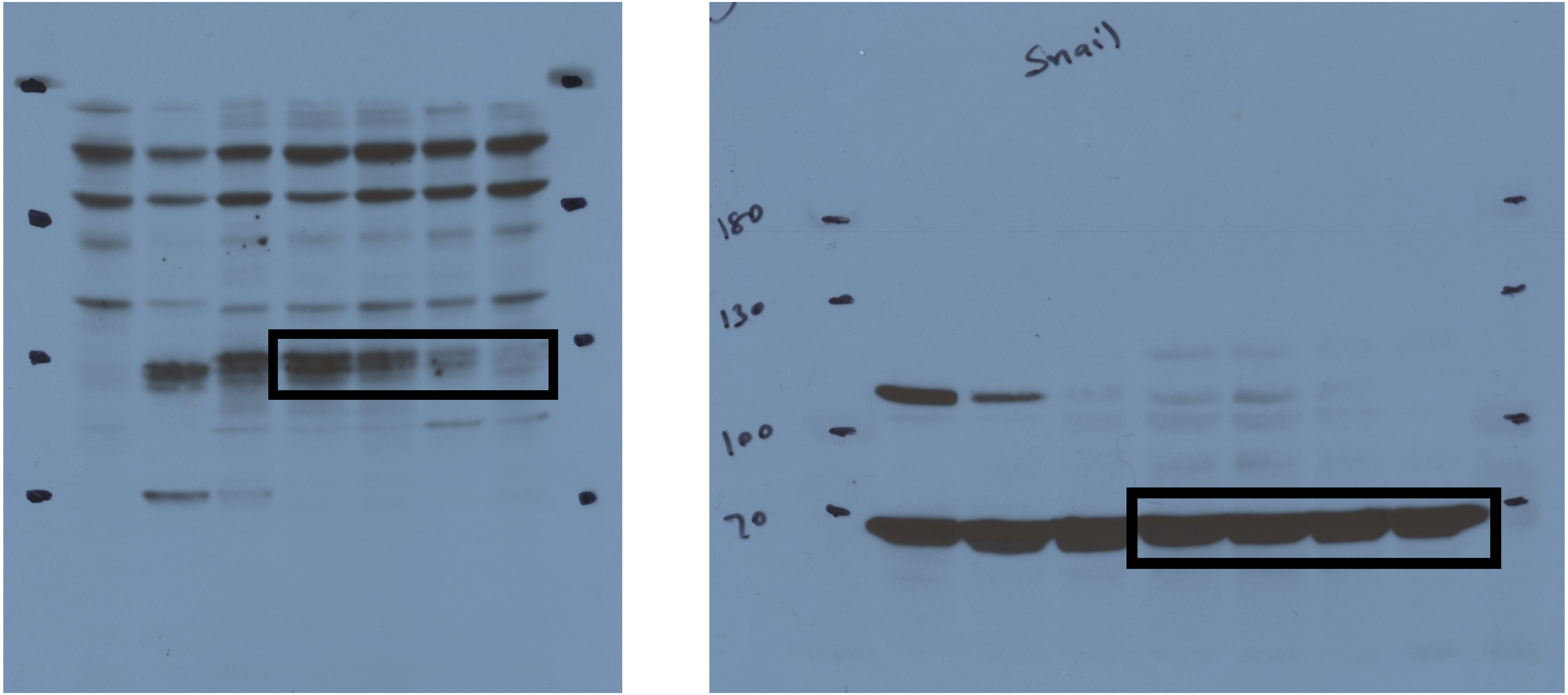

Fig. 5 E

Zeb1

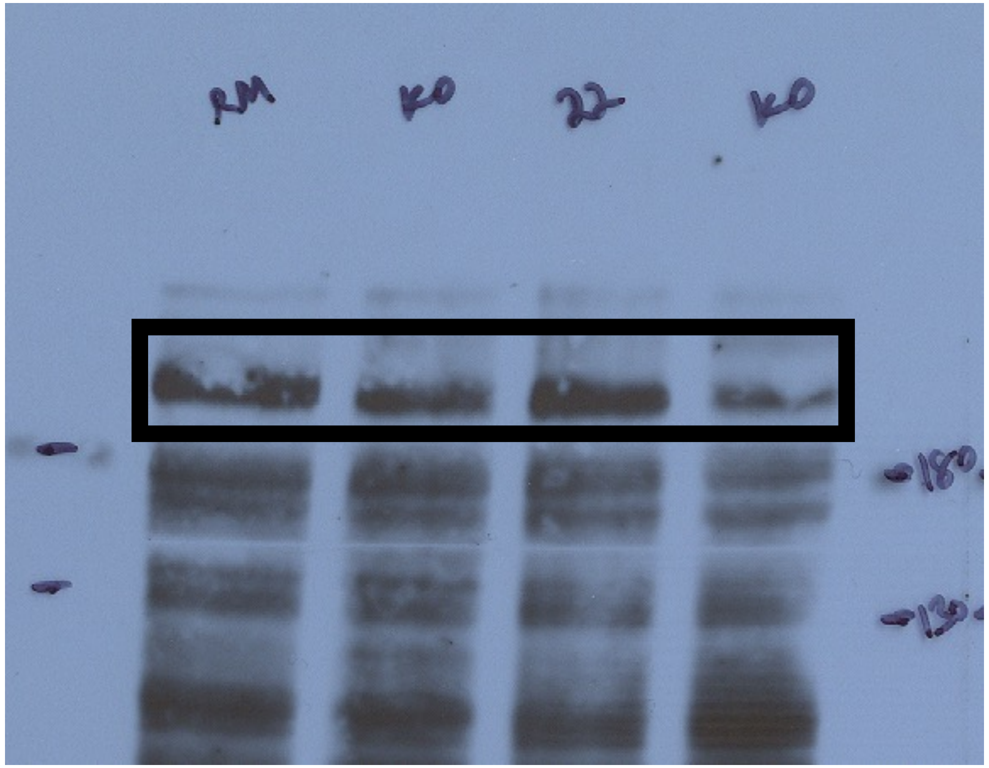

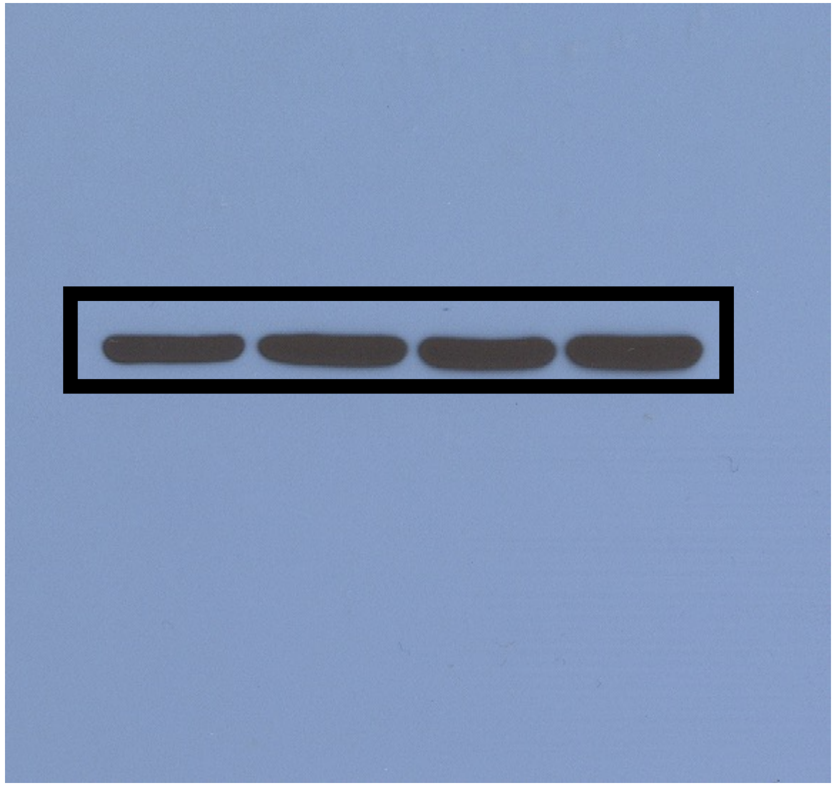

Fig. 5 E

Slug

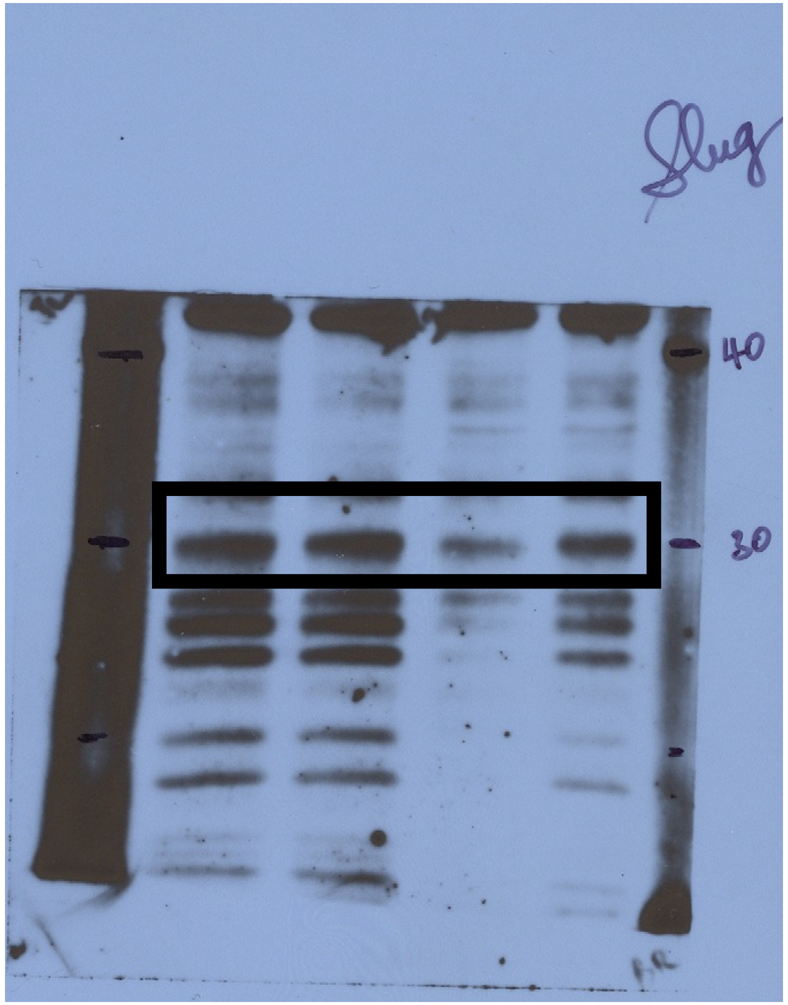

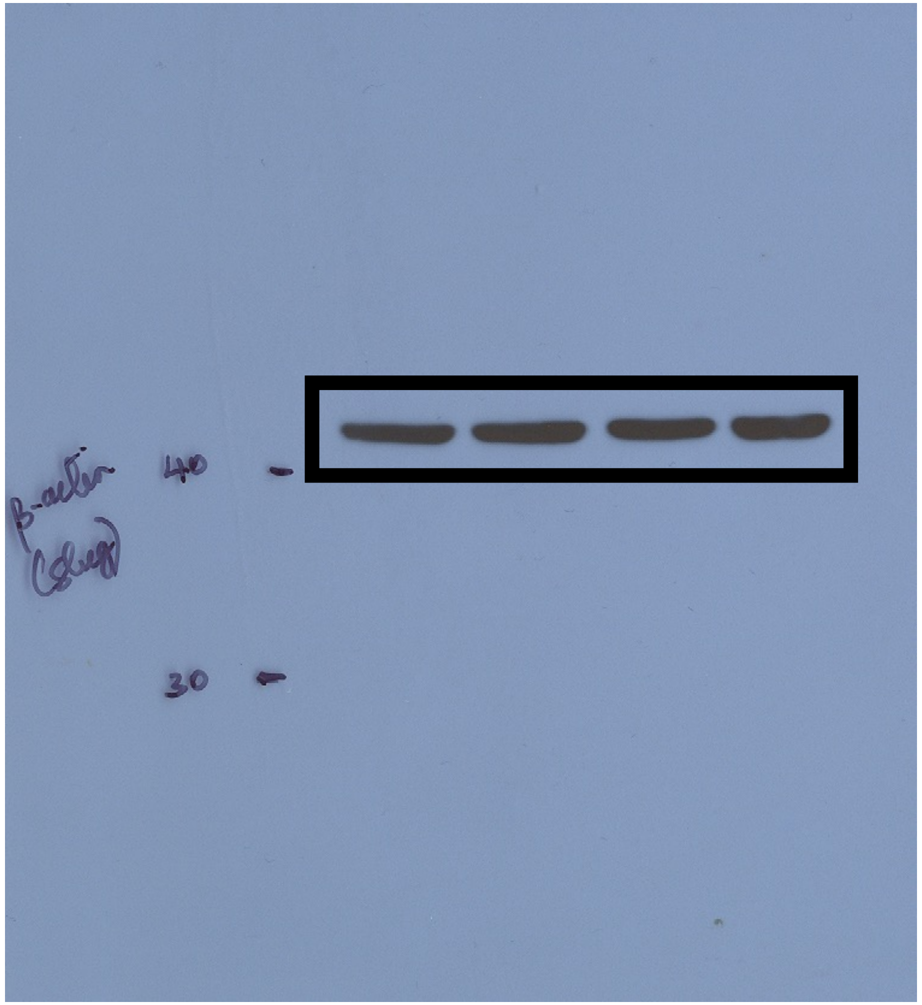

Fig. 5 E

Lin28b

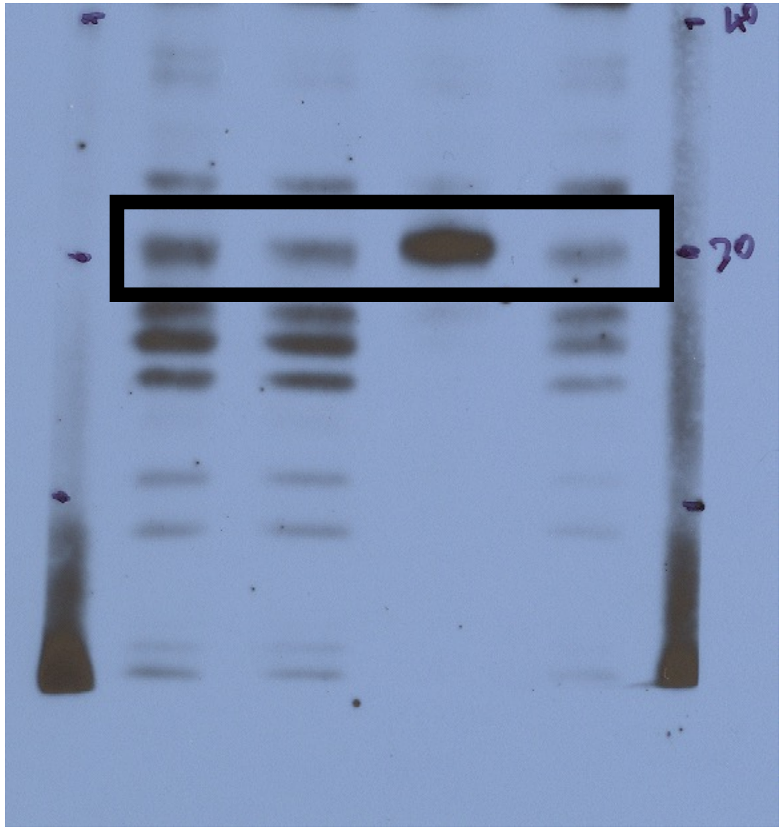

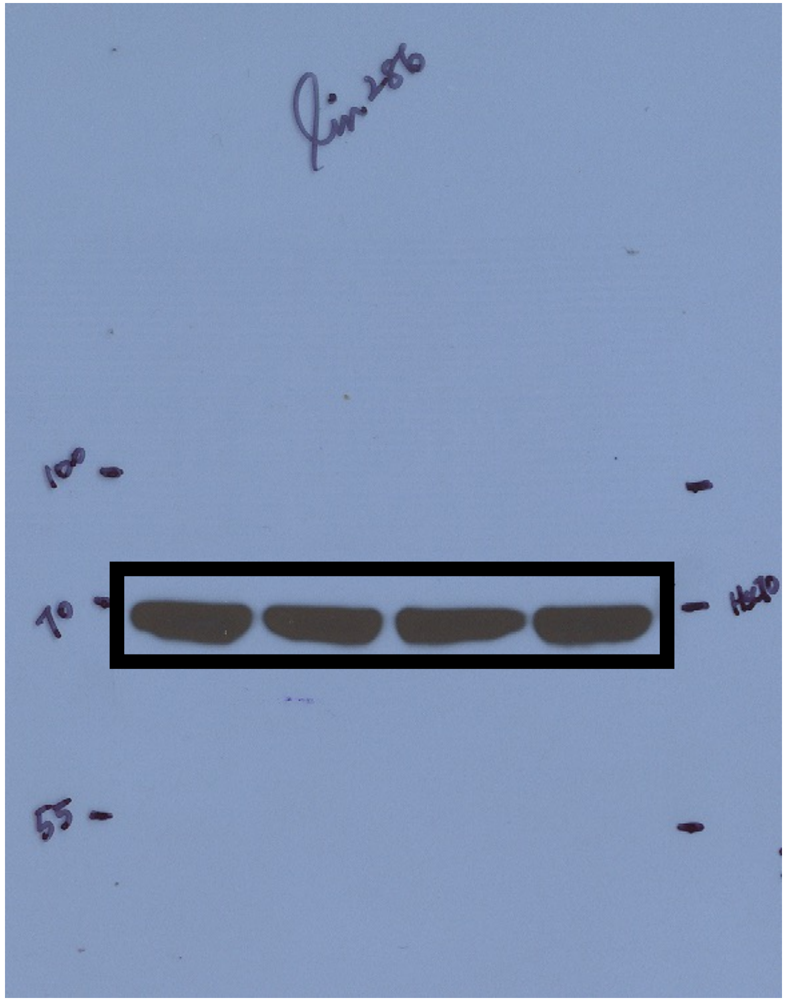

Fig. 5 E

Notch1

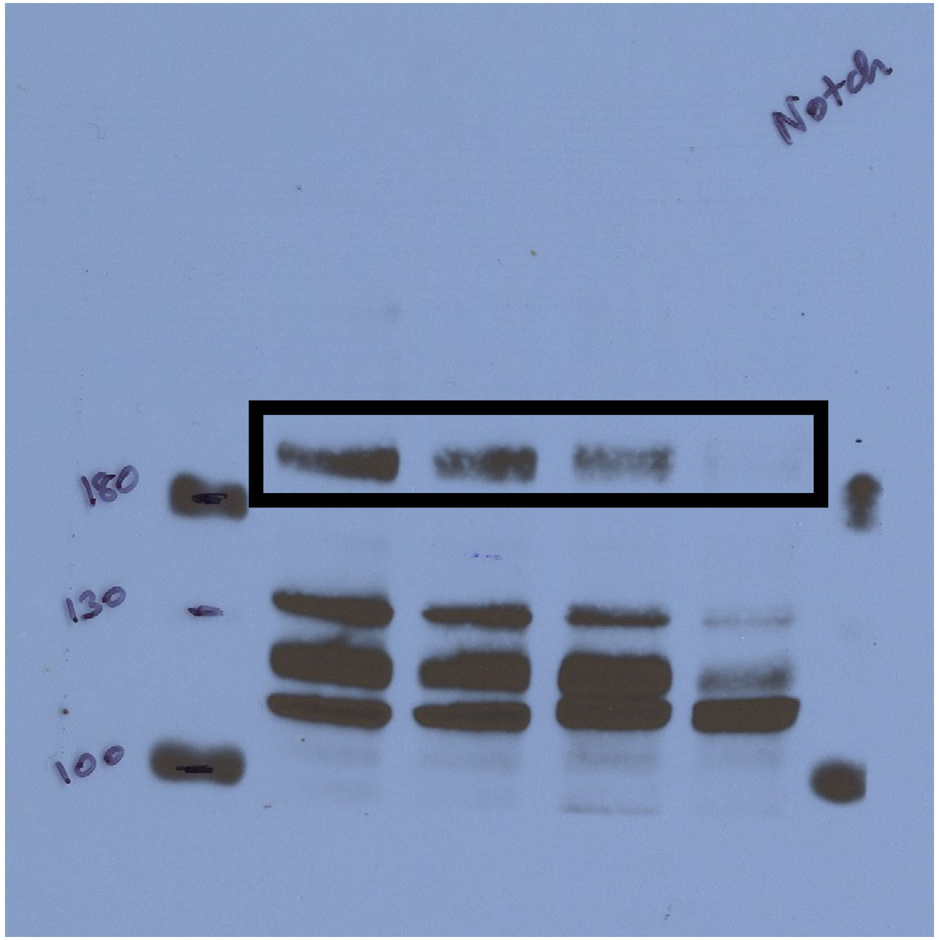

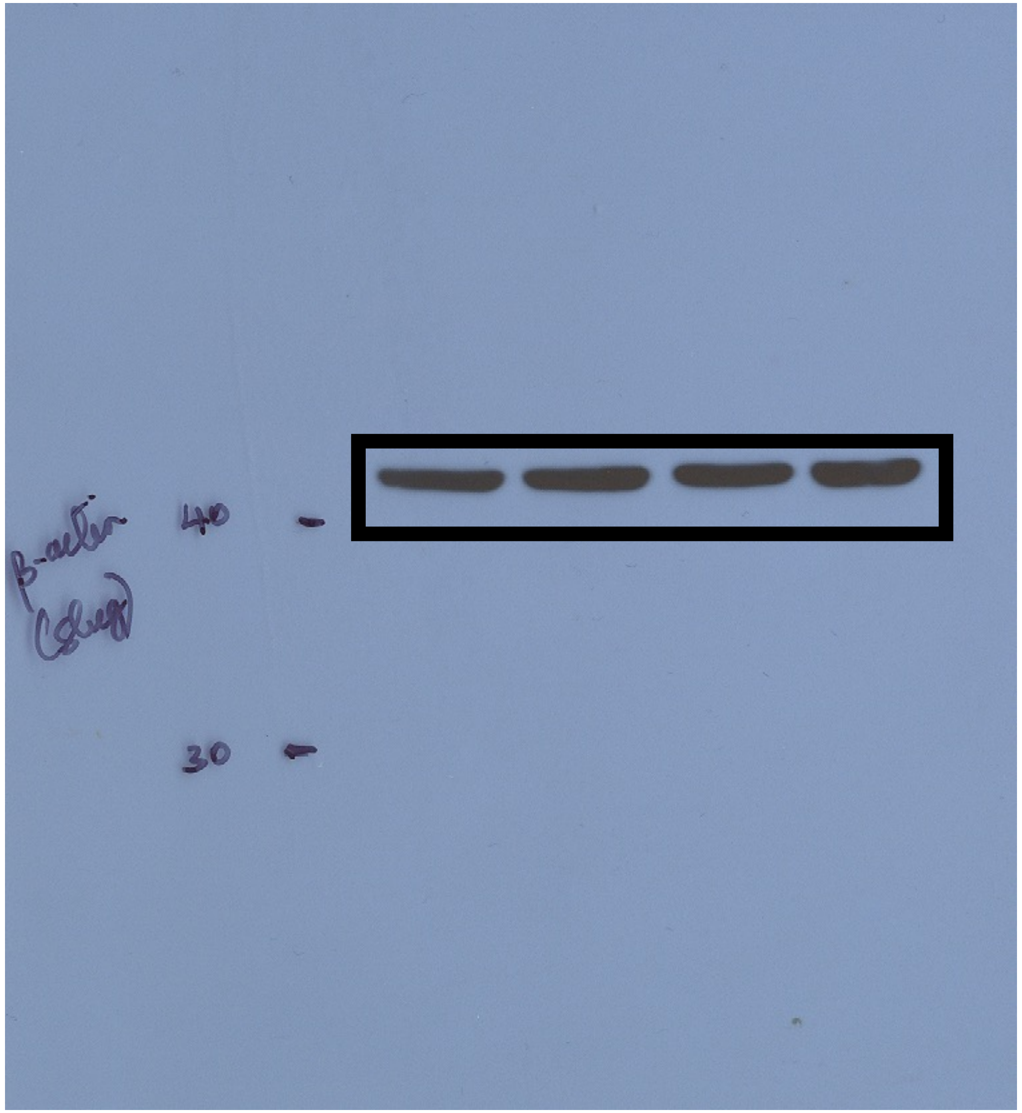

Fig. 5 E

Stat3 - α and β

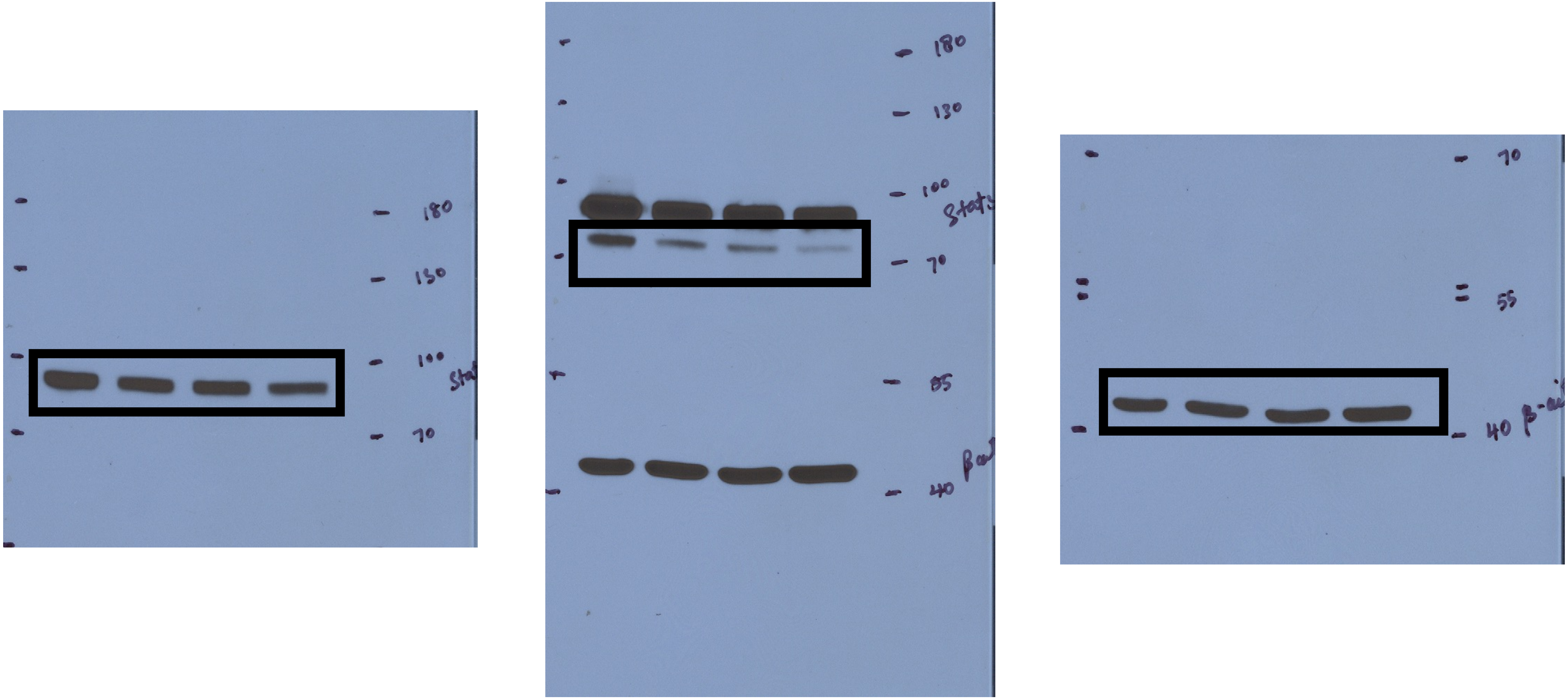

Fig. 5 E

Sox2

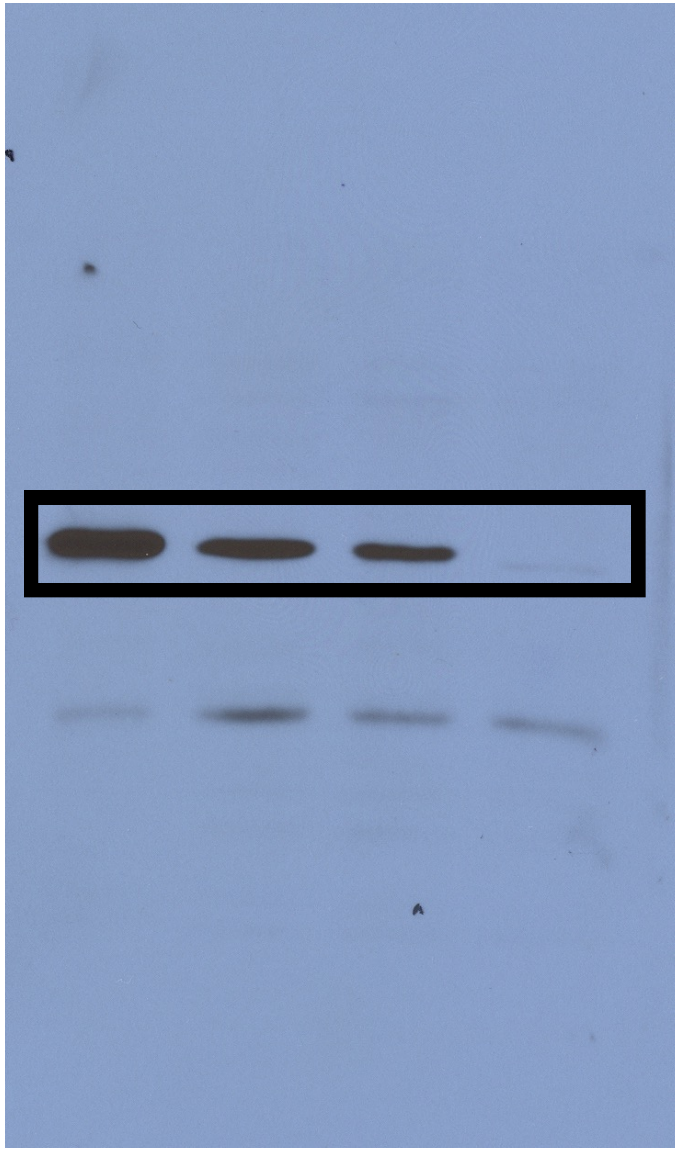

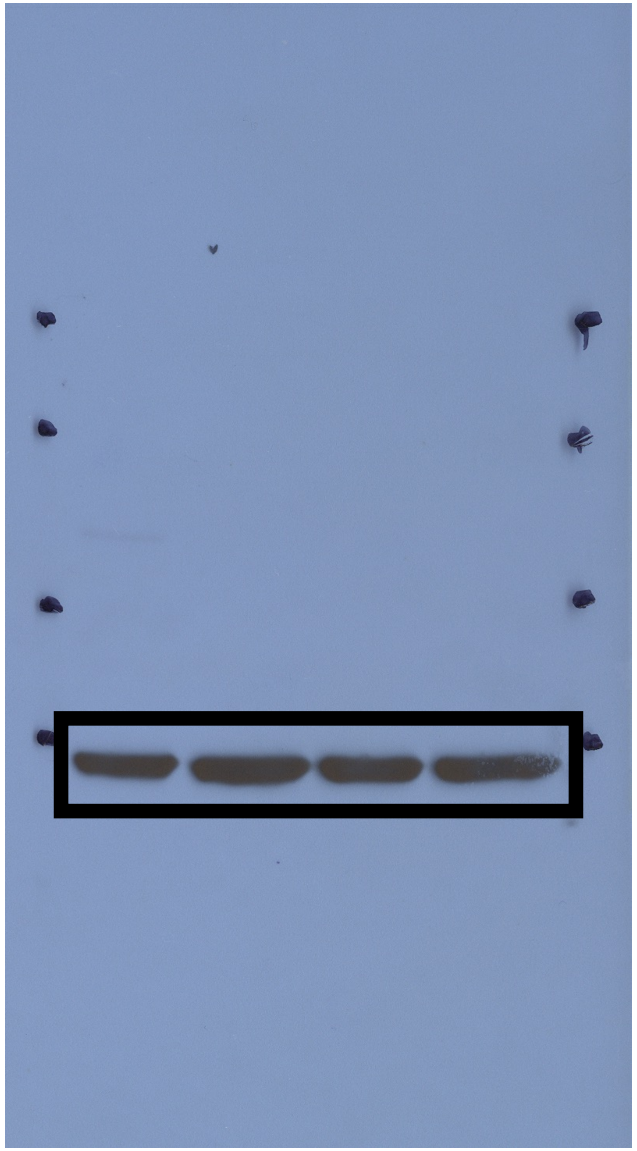

Fig. 5 E

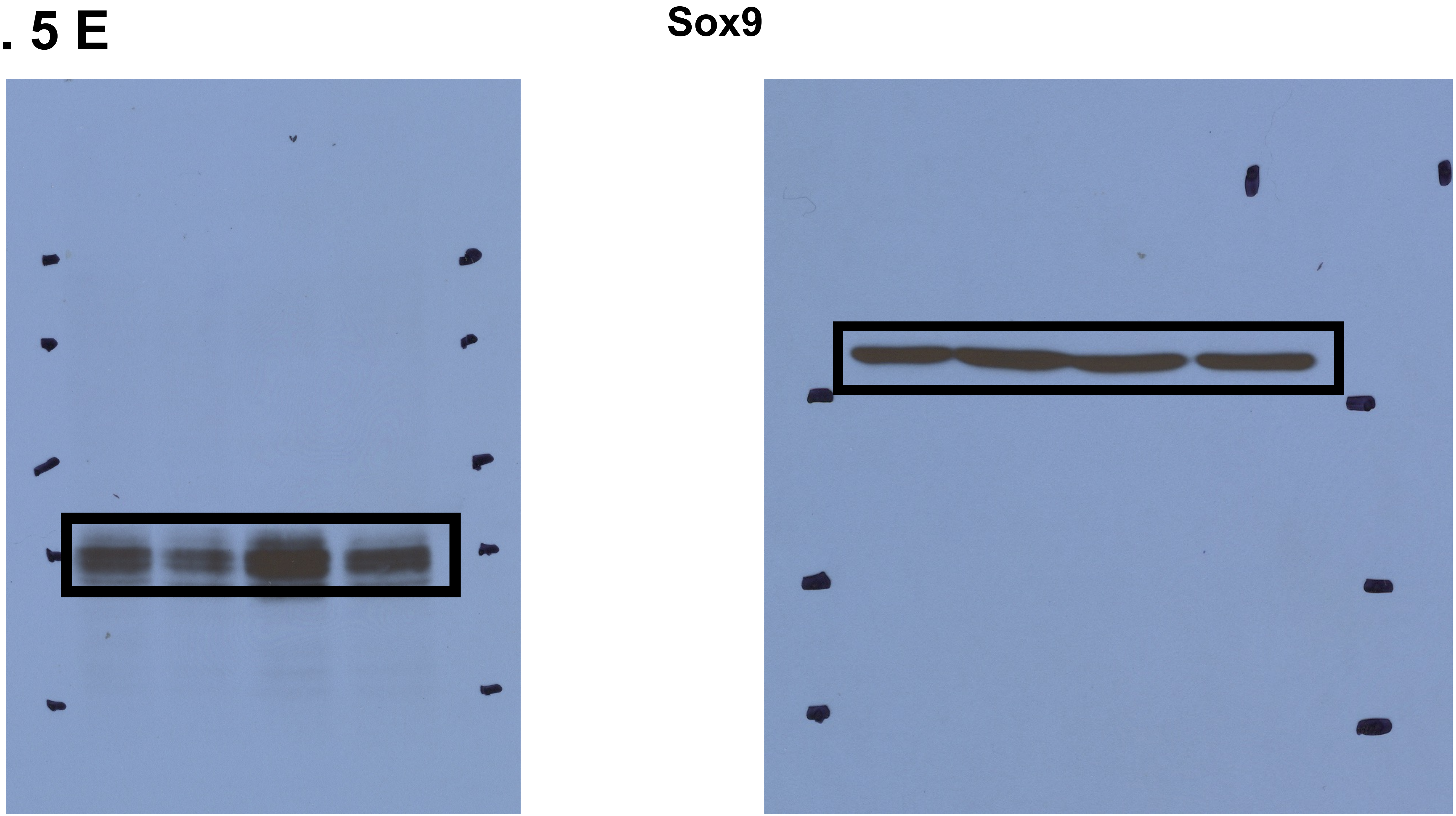

Fig. 5 E

N-Cadherin

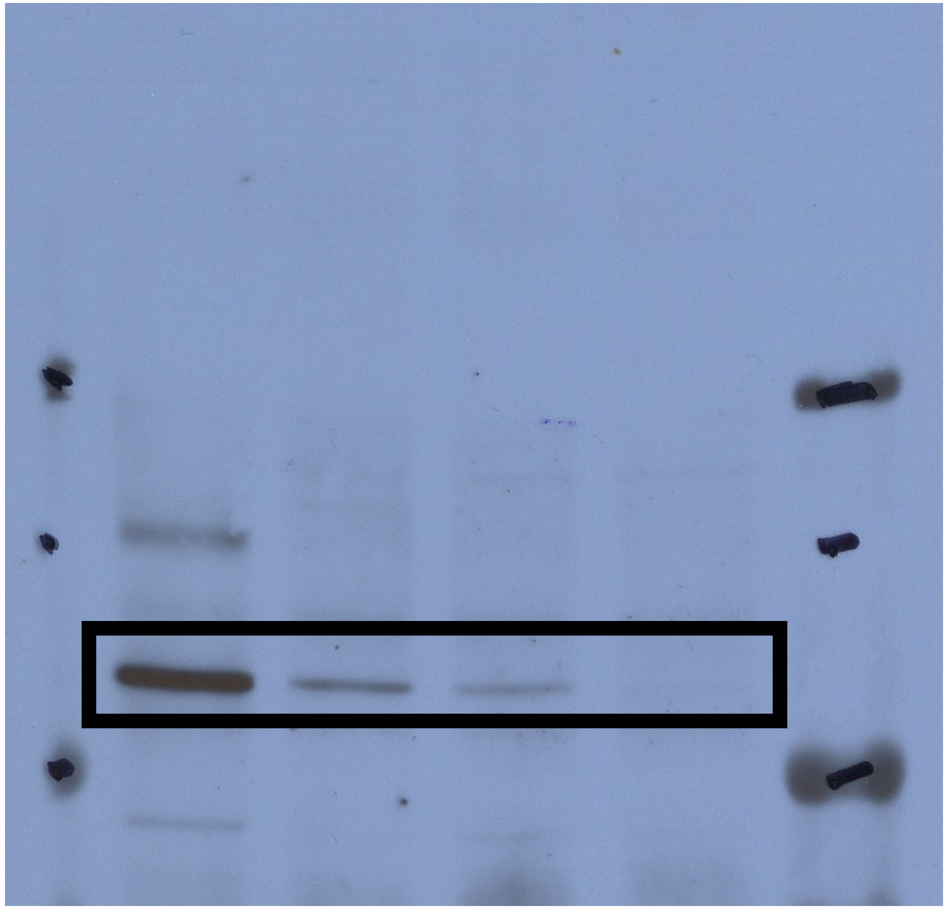

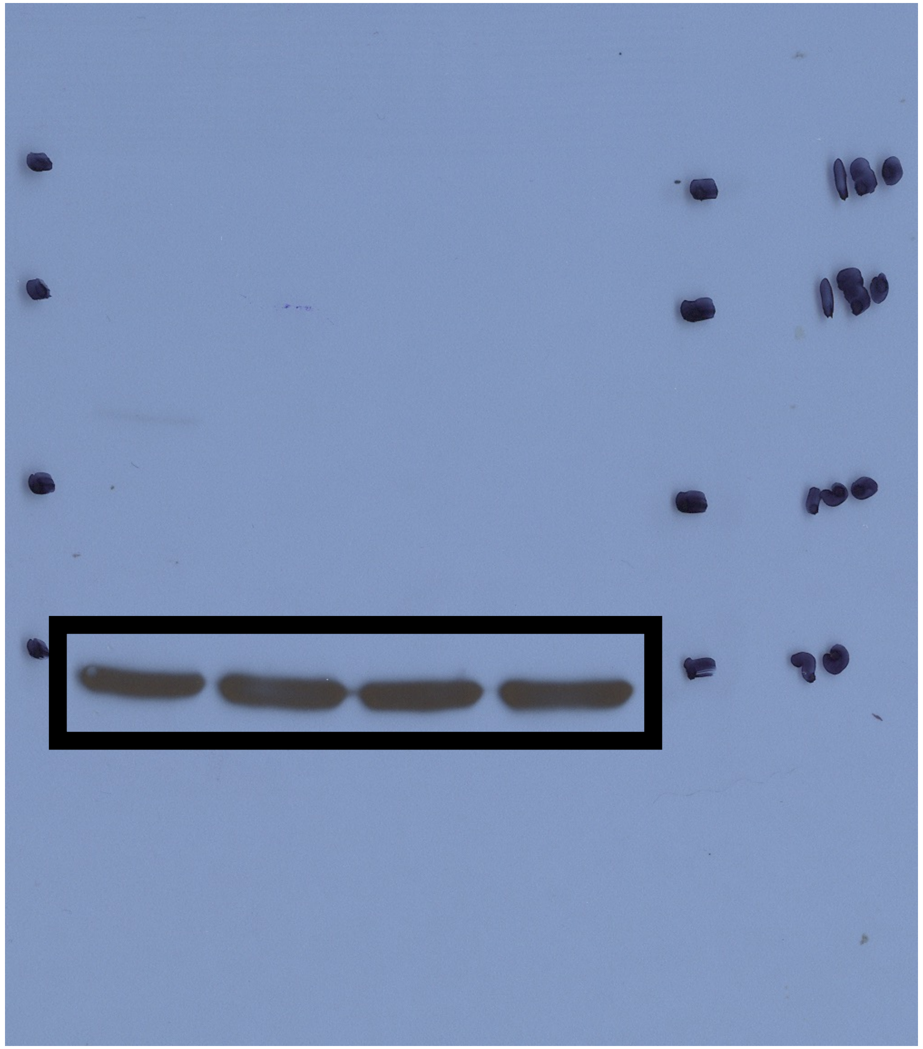

Supplement: Supplementary file 3 — Supplementary Information 3. [file 41598_2024_60052_MOESM3_ESM.pdf]
