# Supplementary material for: GD2 and its biosynthetic enzyme GD3 synthase promote tumorigenesis in prostate cancer by regulating cancer stem cell behavior
Source: Sci Rep. 2024 Jun 12;14:13523. doi: 10.1038/s41598-024-60052-3 (PMC11169677; doi:10.1038/s41598-024-60052-3)

Supplementary Fig. S1A

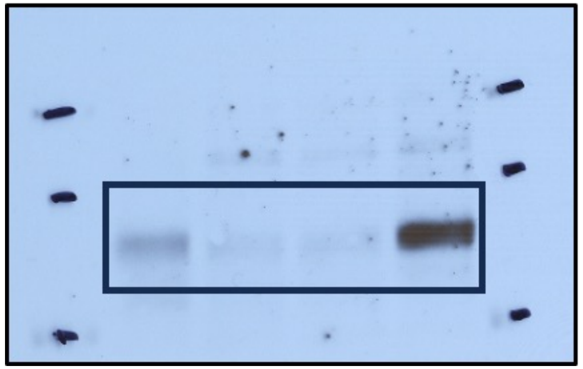

RB1

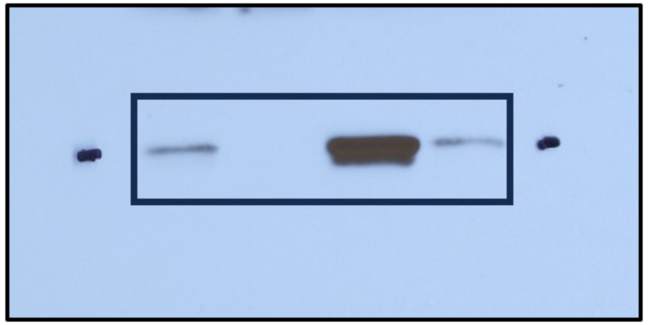

TP53

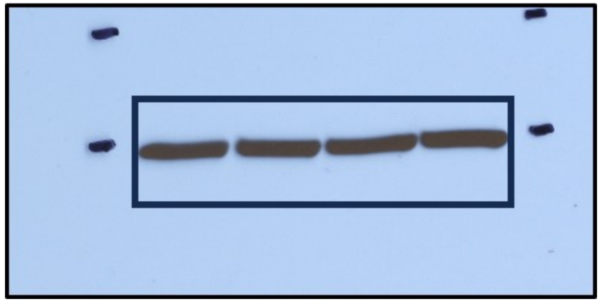

Hsc70

Supplementary Fig. S8A

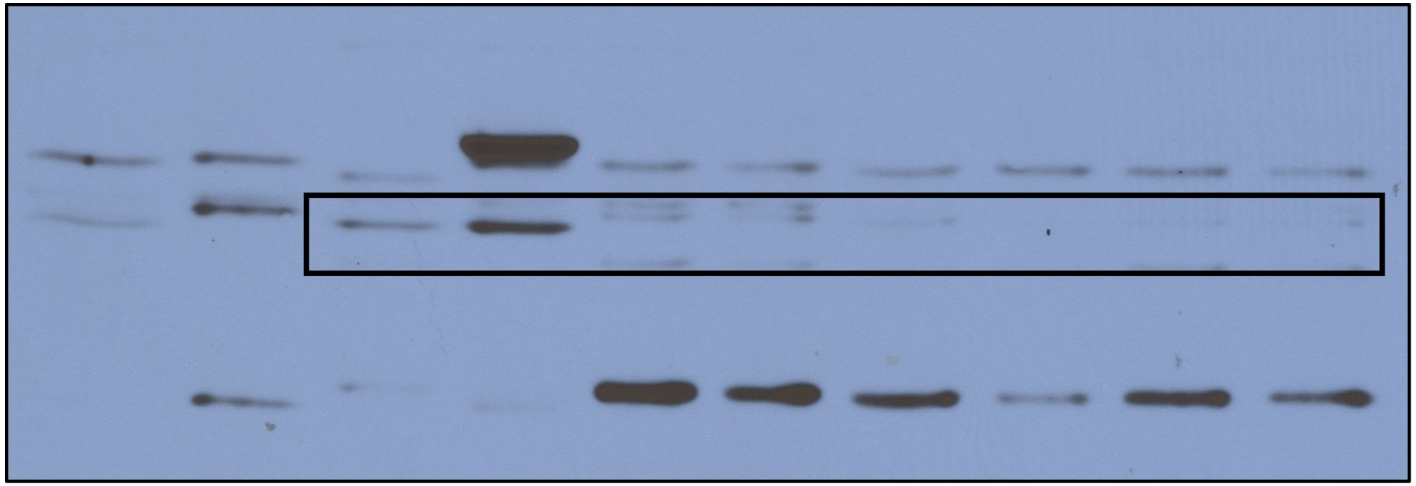

ST8SIA1

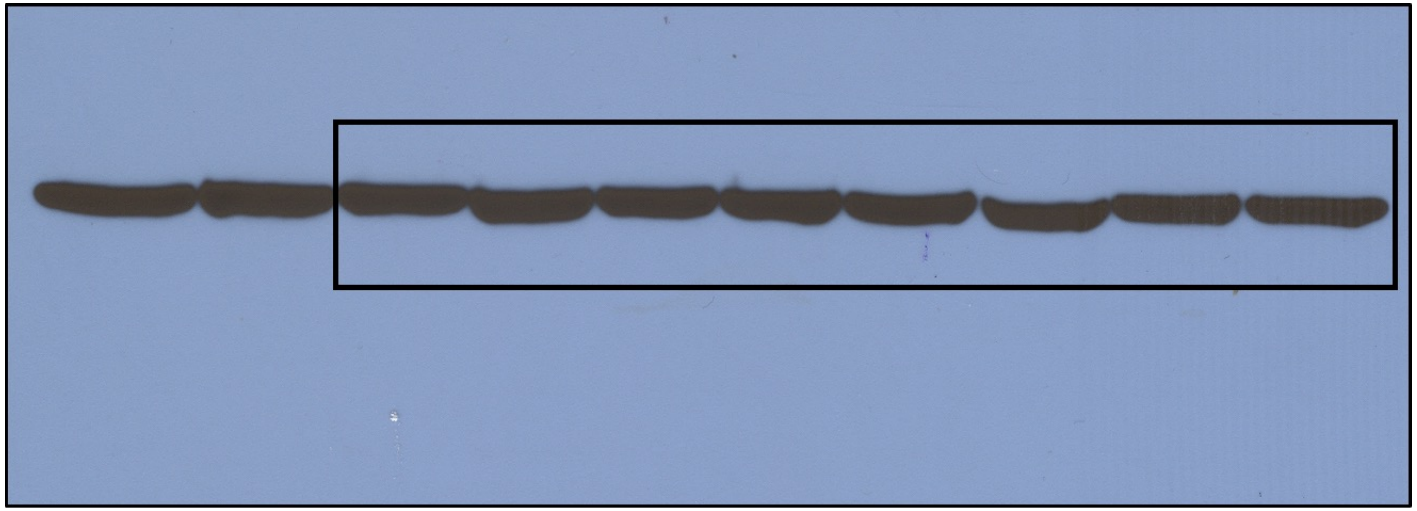

Hsc70

Supplementary Fig. S8C

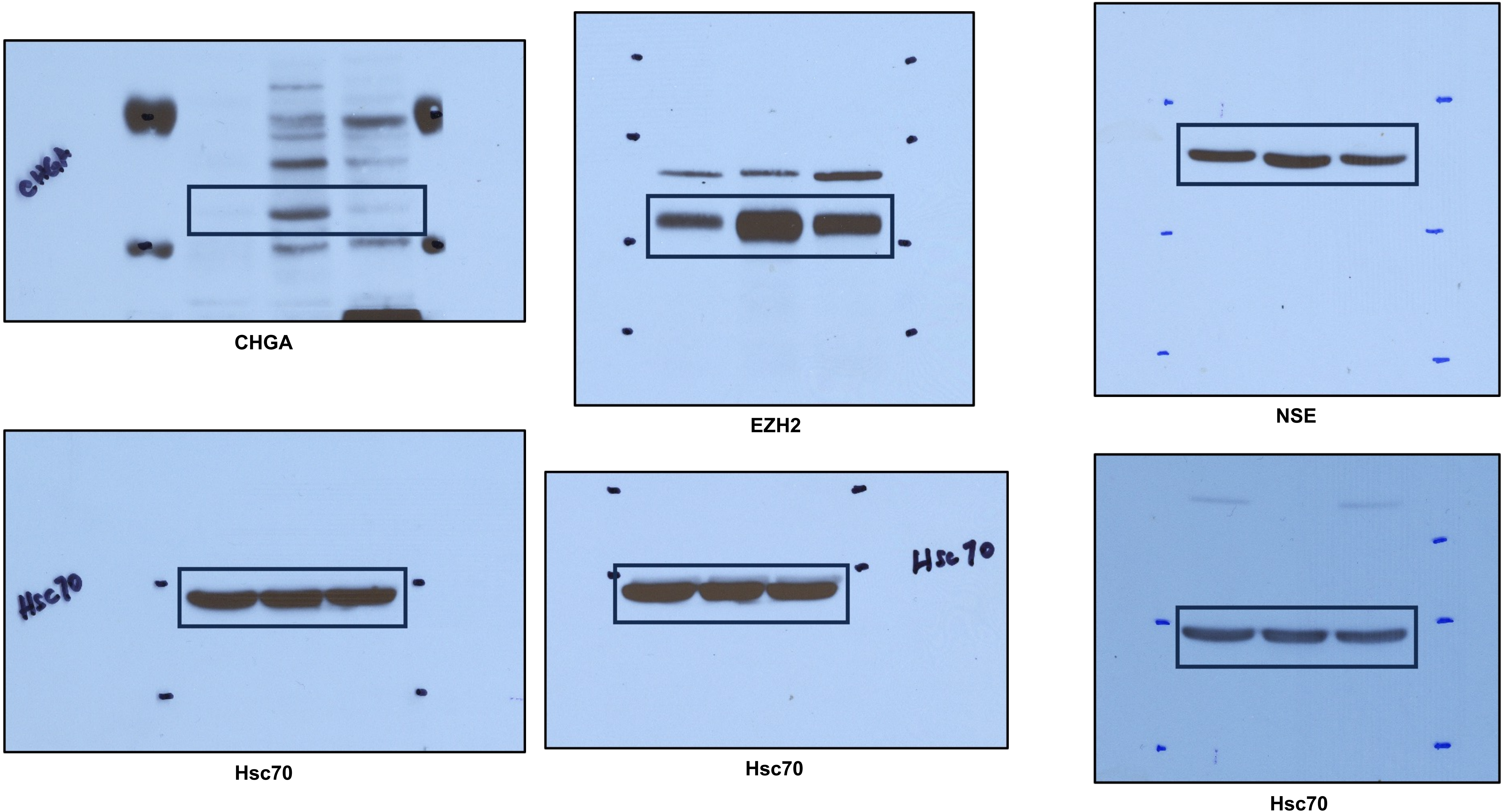

Supplement: Supplementary file 4 — Supplementary Information 4. [file 41598_2024_60052_MOESM4_ESM.pdf]
